# Maternal Obesity in Pregnancy: Risk Factor for Neurodevelopmental Outcomes in Offspring

**DOI:** 10.1111/jnc.70333

**Published:** 2025-12-26

**Authors:** Luisa O. Schmitt, Giuseppe Faraco, Tamires S. Stivanin, Joana M. Gaspar

**Affiliations:** ^1^ Laboratory of Neuroimmune‐Metabolism Federal University of Santa Catarina Florianopolis SC Brazil; ^2^ Graduate Program in Biochemistry Federal University of Santa Catarina Florianopolis SC Brazil

**Keywords:** developmental origins, maternal obesity, neurodevelopment diseases, neuroinflammation, offspring brain

## Abstract

Obesity is a worldwide epidemic disease marked by changes in the function of various tissue and organs, driven by excessive fat accumulation. In recent years obesity was characterized not just by the increase of fat, but also an imbalance of energy homeostasis mechanisms. In parallel with global rise in obesity, the incidence of obesity during pregnancy and lactation had also been steadily increasing. Maternal obesity is a public health issue that affects the child and the mother, in acute and chronic term, being a risk factor for the development of metabolic, hormonal, neurodevelopmental, and psychiatric disorders in offspring. Obesity during the gestation can reprogram the fetal immune, metabolic, endocrine, and neurological systems, influencing offspring's metabolism and mental health. This is supported by the Developmental Origins of Health and Disease (DOHaD) theory, which proposes that environmental factors during critical periods of early development (as the fetal period) can influence the risk of developing diseases later in life. In this review, we focused on how maternal obesity can affect the brain offspring neurodevelopment, neural circuits, synapses, glial cells, and neuroinflammation, which all can influence offspring behavioral disorders.

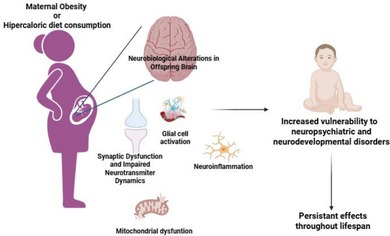

## Graphical abstract

Maternal obesity during pregnancy induces metabolic and inflammatory disturbances that alter the offspring brain environment, characterized by neuroinflammation, glial activation, disrupted neurotransmitter signaling, and mitochondrial and synaptic dysfunction, ultimately increasing the risk of adverse neurodevelopmental outcomes, with persistent effects throughout life.

**Abbreviations**
5‐HIIA: 5‐hydroxyindoleacetic acid
5‐HT: Serotonin
5‐HT1A: Serotonin 1A receptor
ADHD: Attention‐Deficit/Hyperactivity Disorder
AGEs: advanced glycation end products
AgRP: agouti‐related protein
Arc: activity‐regulated cytoskeleton‐associated protein
ASD: autism spectrum disorder
ATP: adenosine triphosphate
BBB: blood–brain barrier
BDNF: brain‐delivered neurotrophic factor
BMI: Body Mass Index
CD: cafeteria diet
CNS: central nervous system
CREB: cyclic AMP response element binding‐protein
CRP: C‐reactive protein
DAT: dopamine transporter
DNA: deoxyribonucleic acid
DNMT1: DNA methyltransferase 1
DOHaD: Developmental Origins of Health and Disease
DRP1: dynamin‐related protein‐1
ED: embryonic day
ER: endoplasmic reticulum
FFA: free fatty acids
GABA: gamma aminobutyric acid
Glu1A: Glutamate Ionotropic Receptor AMPA Type Subunit 1
GluN2B: glutamate [NMDA] receptor subunit epsilon‐2
GLUT: glucose transporters
HFD: high fat diet
HFHS: high fat—high sugar diet
IGF‐1: insulin‐like growth factor 1
IL‐17: interleukin‐17
IL‐1β: interleukin‐1β
IL‐6: interleukin‐6
IRS‐2: insulin receptor substrate 2
JAK: Janus kinase
MCP‐1: monocyte chemoattractant protein 1
Mfn‐2: mitofusin‐2
MIA: maternal immune activation
NAc: nucleus accumbens
NFkB: nuclear factor kappa‐light‐chain‐enhancer of activated B cells
NGF: nerve growth factor
NMDA: N‐methyl D‐aspartate receptor
NPC: neuronal precursor cells
NPY: neuropeptide Y
OXTR: oxytocin receptor
pAMPK: phosphorylated AMP‐activated protein kinase
PD: postnatal day
PFC: prefrontal cortex
POMC: pro‐opiomelanocortin
PSD‐95: postsynaptic density 95
ROS: reactive oxygen species
SERT: serotonin transporter
SOCS3: suppressor of cytokine signaling 3
SOD: superoxide dismutase
TLR4: toll‐like receptor 4
TNF‐α: tumor necrosis factor‐α
TPH2: tryptophan hydroxylase 2
VGLUT1: vesicular glutamate transporter 1
VTA: ventral tegmental area
WHO: World Health Organization
WSD: western‐style diet
α‐MSH: α‐melanocyte‐stimulating hormone

## 1 Introduction

Obesity is a chronic metabolic disorder characterized by altered tissue and organ function due to excessive adiposity and persistent low‐grade inflammation, driven largely by adipose tissue–derived cytokines that disrupt systemic metabolism (Rubino et al. 2025; World Obesity Federation, W. heath organization 2023). Rather than a passive result of fat accumulation, obesity reflects dysregulation of energy homeostasis involving neuroendocrine, immune, and metabolic circuits (Schwartz et al. 2017). The excessive accumulation of adipose tissue leads to a range of metabolic, inflammatory, and biochemical perturbations, which collectively increase the susceptibility to a diverse spectrum of comorbid conditions such as type 2 diabetes, cardiovascular disease, and neuropsychiatric disorders (Lavie et al. 2015; WHO 2025).

The rising prevalence of obesity among women of reproductive age extends these metabolic perturbations to gestation and lactation, where maternal obesity alters the intrauterine environment and affects fetal organogenesis, including brain development (WHO Europe 2022; Barker 1995; Wadhwa et al. 2009). Maternal obesity–induced inflammation, hormonal imbalance, oxidative stress, mitochondrial dysfunction, and epigenetic modifications can disrupt neurodevelopmental trajectories, increasing the risk of cognitive and behavioral impairments in offspring (Contu and Hawkes 2017; Davis and Mire 2021; Huang et al. 2014; Kong et al. 2020; Page et al. 2019; Weber‐Stadlbauer 2017).

This narrative review examines mechanistic pathways linking maternal obesity to offspring neurodevelopmental, metabolic, and behavioral alterations, emphasizing inflammatory, mitochondrial, and synaptic dysfunction as key mediators. PubMed was used as the primary database for our bibliographic search. The selected keywords included: “maternal high‐fat diet” or “maternal obesity” and “offspring adipose tissue” or “offspring brain” or “offspring neuroinflammation” or “offspring mitochondria” or “offspring brain circuits” or “offspring hypothalamus” or “offspring hippocampus” or “offspring synapses” or “offspring glia” or “offspring behavior” or “offspring psychiatric disorder”.

## 2 Obesity as Inflammatory Disease and Its Influence on Fetal Neuroinflammation

Obesity is characterized by excessive and abnormal white adipose tissue accumulation. White adipose tissue has important function in the storage of triglycerides, and releasing them as free fatty acids during energy demand. Additionally, it acts as an endocrine organ, secreting hormones and cytokines that regulate metabolism, inflammation, and insulin sensitivity (Rosen and Spiegelman 2014). During the progression of overweight and obesity, adipocytes increase in number and in weight, creating zones of hypoxia, which are essential for the recruitment and activation of macrophages, as well as to the release of proinflammatory cytokines, leading to the cell dysfunction and consequently insulin resistance (Elias et al. 2012; Frayn and Karpe 2014).

The inadequate maternal nutrition and obesity during gestation induces significant metabolic changes, such as endocrine imbalance, increase in proinflammatory cytokines levels, and stress that can impair fetal brain development, and metabolic programming, increasing the risk of chronic diseases later in life (Heerwagen et al. 2010; Şanlı and Kabaran 2019). The maternal immune activation (MIA) hypothesis suggests that immune system activation in utero can influence the development of neural circuits, leading to several neurodevelopmental conditions, and psychiatry diseases (Gilmore and Jarskog 1997; Patterson 2002). The first evidence supporting this hypothesis came from studies on maternal exposure to viral infections—such as influenza, herpes simplex virus type 2, rubella, and cytomegalovirus—which increase maternal cytokine levels and elevate the risk of schizophrenia in the child (Brown et al. 2009; Brown et al. 2004; Buka et al. 2008). Taking this into account the increase in the proinflammatory cytokines occurring in obesity can modulates fetal development.

Several studies in humans, have demonstrated and increased in inflammatory markers in women with obesity, as well as in the placenta and cord‐blood. In the blood of pregnant women with obesity (Table 1), it has been described that occurs an increase in tumor necrosis factor‐α (TNF‐α), interleukin (IL)‐1β, IL‐17, IL‐6, among other cytokines (Jancsura et al. 2023; Stewart et al. 2007), as well as an increase in the gene expression of inflammatory genes on cord‐blood of offspring compared with the pregnant women with normal weight (Dosch et al. 2016). Pregnant women with overweight have higher levels of serum C‐reactive protein (CRP) and monocyte chemoattractant protein 1 (MCP‐1) at second‐trimester than pregnant woman with normal weight (Gaillard et al. 2016; Madan et al. 2009). Mothers with obesity or overweight have increased expression of those genes in their blood, proposing the maternal inflammatory condition can arrive by circulation and impact intrauterine environment (Nakandakare et al. 2021). Newborns from mothers with overweight and obesity have a higher expression of inflammation genes, TNF‐α, nuclear factor kappa‐light‐chain‐enhancer of activated B cells (*NFkB*) and Toll‐like receptor 4 (*TLR4*), in the cord‐blood than newborns from women with normal weight (Nakandakare et al. 2021). Another important aspect is the increased macrophage population on the placenta of women with obesity compared to pregnant women with normal weight, characterized by increased CD14^+^, CD68^+^, and CD11b^+^ markers (Challier et al. 2008), as well as increased gene expression of cytokines IL‐1, TNF‐α, IL‐6. Fetoplacental immune activation also has sex‐specific alterations (Leon‐Garcia et al. 2015; Shook et al. 2023). Placenta from male fetus of mothers with obesity have higher density of Hofbauer cells (placental resident macrophage) than female fetus placenta from mothers with obesity, suggesting maternal obesity is linked to sex‐specific changes in Hofbauer cells phenotype, characterized by increased cell circularity and hyperplasia in male placenta (Shook et al. 2023).

**TABLE 1:** Human studies.

References	Maternal model	Analyzed tissue	Main results
Jancsura et al. (2023)	Pregnant woman with obesity	Maternal plasma	↑TNF‐α, ↑IL‐1β, ↑IL‐17, ↑IL‐6
Stewart et al. (2007)	Pregnant woman with obesity	Maternal plasma	↑IL‐6
Dosch et al. (2016)	Pregnant woman with obesity	Cord‐blood	↑TNF‐α, ↑CRP
Gaillard et al. (2016)	Pregnant woman with obesity	Maternal plasma	↑CRP
Madan et al. (2009)	Pregnant woman with obesity	Maternal serum	↑CRP, ↑MCP‐1
Nakandakare et al. (2021)	Pregnant woman with obesity	Maternal blood and cord‐blood	↑TNF‐α, ↑NFkB, ↑TLR4 (gene expression)
Challier et al. (2008)	Pregnant woman with obesity	Placenta	↑IL‐1, ↑TNF‐α, ↑IL‐6, ↑CD14^+^, ↑CD68^+^, ↑CD11b^+^
Shook et al. (2023)	Pregnant woman with obesity	Placenta	↑Density of Hofbauer cells
Hirschmugl et al. (2021)	Pregnant woman with obesity	Placenta	↑Clearence of free fatty acids
Costa et al. (2016)	Pregnant woman with obesity	Cord‐blood	↑Total free fatty acids, ↑Palmitate, ↑Stearate
Malti et al. (2014)	Pregnant woman with obesity	Maternal and offspring plasma	↑Triglycerides, ↑Cholesterol
Santos‐Rosendo et al. (2020)	Pregnant woman with obesity	Placenta	↓Superoxide dismutase activity, ↓catalase activity, ↑nitrotyrosine residues
Ou et al. (2015)	Pregnant woman with obesity	Offspring brain	↓Cerebral white matter integrity
Koning et al. (2017)	Pregnant woman with obesity	Offspring brain	↓Cerebelar growth at pregnancy
Spann et al. (2020)	Pregnant woman with obesity	Offspring brain	↑Left thalamus connectivity, ↓Frontothalamic connectivity
Li et al. (2021)	Pregnant woman with obesity	Offspring	Increased risk of ADHD in offspring.

Dysregulated or persistent neuroinflammation can contribute to the development of neurological conditions (Ransohoff et al. 2015) (Guillemot‐Legris and Muccioli 2017; Han et al. 2021). When discussing the MIA hypothesis, elevated maternal cytokine levels can reach fetus and impact the fetal brain development, not only affecting blood–brain barrier formation and neurodevelopment but also contributing to persistent Blood Brain Barrier (BBB) dysfunction (Zhao et al. 2022). The chronic consumption of high fat diet (HFD) by pregnant nonhuman primates (Table 2) can increase proinflammatory cytokines and activate microglia cells on fetuses, suggesting that maternal HFD consumption can impact the fetal neurodevelopment (Grayson et al. 2010). Additionally, at 12 weeks of age, offspring of mothers fed a HFD exhibited elevated hypothalamic levels of IL‐6, IL‐1β, and TNF‐α, indicating the presence of hypothalamic inflammation (Ornellas et al. 2016). Table 3 summarizes the studies in rodent animal models of HFD induced obesity in inflammatory changes in the offspring.

**TABLE 2:** Summary of experimental studies conducted in nonhuman primate models of obesity.

References	Model	Tissue	Main results
Dunn et al. (2022)	Nonhuman primates fed with HFD	Offspring basolateral amygdala	↑Microglia count, microglia number was correlated with maternal adiposity
Grayson et al. (2010)	Nonhuman primates fed with HFD	Offspring hypothalamus	↑IL‐1β, ↑IL‐1, ↑Iba1
Sullivan et al. (2010)	Nonhuman primates fed with HFD	Offspring rostral raphe	↑TPH2, ↑5‐HT1A; ↑Anxiety in response to threatening novel objects
Thompson et al. (2018)	Nonhuman primates fed with HFD	Offspring behavior	↑Reactive anxiety, ↑Ritualized anxiety

**TABLE 3:** Summary of experimental studies conducted in rodent models of obesity.

References	Maternal model	Offspring tissue	Main results
Hatanaka et al. (2016)	Mice fed with HFD	Somatosensory cortex	synaptic instability and progressive spine loss
Maldonado‐Ruiz et al. (2019)	Wistar rats fed with CD	Hypothalamus	↑Iba1, ↑c‐Fos, ↑Microglial activation
Ojeda et al. (2023)	Mice fed with HFD	Hippocampus	↑Microglia and astrocytes density
Bordeleau et al. (2020)	Mice fed with HFD	Hippocampus	↑Microglia interaction with astrocytes, changed microglia morphology
Shiadeh et al. (2024)	Mice fed with HFD	Hippocampus	↑Microglia density and microglia soma, ↓PSD‐95
Bordeleau et al. (2021)	Mice fed with HFD	Hippocampus	↓Mature lysossomes inside microglia, ↑Microglia interaction on synaptic contact, ↓Igf1, changed myelin organization
Mendoza‐Romero et al. (2025)	Mice fed with HFD	Hypothalamus	↑Interaction between microglia and AgRP neurons
Valdearcos et al. (2025)	Mice fed with HFD	Hypothalamus	↑Reactive microglia, ↑Microglia phagocity activity, ↑Internalized PSD‐95 inside microglia
Bordeleau et al. (2022)	Mice fed with HFD	Hippocampus	↑Microglia‐blood vessel proximity, ↑Microglia‐synapses interaction
Kim et al. (2016)	Mice fed with HFD	Hypothalamus	↑Astrocytes proliferation and number
Kjaergaard et al. (2017)	SD rats treated with CAF diet	Hypothalamus	↑Hypothalamic astrogliosis
Huang et al. (2024)	Mice fed with HFD	Hypothalamus	↑Astrocytes interaction with AgRP/NPY neurons
Poon et al. (2012)	SD rats fed with HFD	Hypothalamus	↑NPY protein and positive neurons
Stachowiak et al. (2013)	SD rats fed with HFD	Hypothalamus	↑AgRP/NPY neurons, ↓α‐MSH
Ornellas et al. (2016)	Mice fed with HFD	Hypothalamus	↑NPY, ↓POMC, ↓α‐MSH (proteins), ↑IL‐6, ↑IL‐1β, ↑TNF‐α.
Desai et al. (2016)	SD rats fed with HFD	Hypothalamus	At PD‐1 ↓mTOR, ↓pAMPK, ↓DNMT1. At 6 months of age ↑AgRP/NPY, ↓POMC
Park et al. (2020)	Mice fed with HFHS	Hypothalamus	ER stress on hypothalamic neurons
Cardenas‐Perez et al. (2018)	Wistar rats fed with HFD	Hypothalamus	Failure in glucose, leptin and insulin sensitivity, ↑mitochondrial‐ER interaction, ↑mitochondrial fusion
Dearden et al. (2020)	Mice fed with HFD	Hypothalamus	↓Bub1b, ↓Ki67, ↓Pcna (proliferative genes)
Kulhanek et al. (2022)	Mice fed with HFD	Hypothalamus	Changed transcriptomic profile, mainly oxidative phosphorylation genes.
Mizera et al. (2022)	Wistar rats fed with HFD	Hippocampus	↑Extracellular glutamate, ↑VGLUT1, ↑GluN2B
Lin et al. (2021)	SD rats fed with HFD	Hippocampus	GlutA1 hyperpalmitoylation, central insulin resistence
Page et al. (2014)	SD rats fed with HFD	Hippocampus	↓BDNF, ↓Arc, ↓NGF, ↓NR2B, ↓synaptophysin, ↑synaptotagmin
Yan et al. (2017)	Mice fed with HFD	Hippocampus	↓BDNF, ↓CREB, ↓Grin2b, ↑DNA methyltransferase
Curi et al. (2021)	Mice fed with HFD	Hippocampus	↓BDNF, ↓5‐HT1A, ↑Mash1
Gonçalves et al. (2017)	Wistar rats fed with HFD	Hippocampus	↑Histone H4 acetylation at PD21, ↓Histone H4 acetylation at PD50
Glendining and Jasoni (2019)	Mice fed with HFD	Hippocampus	↑binding of H3 acetylation at OXTR in male pups and ↓OXTR promoter H3 methylation in female pups
Vucetic et al. (2010)	Mice fed with HFD	VTA, NAc and PFC	↑DAT, ↓dopamine receptor D1 and D2, ↓DARPP‐32
Lippert et al. (2020)	Mice fed with HFD	Striatum	Silenced dopaminergic midbrain neurons, ↓synaptic connectivity, ↓dopamine release
Dias‐Rocha et al. (2023)	Wistar rats fed with HFD	NAc	In male pups ↑DAT, ↓dopamine receptor D2. In female pups‐↑DARPP‐32
Peleg‐Raibstein et al. (2012)	Mice fed with HFD	Hippocampus	↑BDNF, ↑GABAA alpha2 receptor, ↑5‐HT1A
Paradis et al. (2017)	SD rats fed with HFHS	Hypothalamus, Nac and VTA	↓Hypothalamic GABAA alpha5 subunit, ↑Nac and VTA GABAA alpha1
Dias et al. (2020)	Mice fed with HFD	Hippocampus	↑TPH2, ↑BDNF, ↑pJNK, ↑TNF‐α.
Moreton et al. (2019)	Wistar rats fed with CD	PFC	↑5‐HIIA, ↑5‐HIIA / 5‐HT ratio
Gawlińska et al. (2021)	Mice fed with HFD	PFC, NAc and striatum	↓5‐HT2C at PD28, ↑5‐HT2C at PD63
Sasaki et al. (2014)	Long Evans rats fed with HFD		↓Anxiety‐like behavior
Sivanathan et al. (2015)	Long Evans rats fed with HFD		↑Anxiety‐like behavior
Wu et al. (2013)	Wistar rats fed with HFD		Impaired reversal learning
Graf et al. (2016)	Mice fed with HFD		↓Time spent exploring the new object
Kang et al. (2014)	Mice fed with HFD		In females ↑anxiety, ↓sociability. In males ↑Hiperactivity
Buffington et al. (2016)	Mice fed with HFD		↓Interaction with other mice, leading to social deficit
Peleg‐Raibstein et al. (2016)	Mice fed with HFD		↑Hedonic‐like behavior to abuse drugs and palatable food

In addition to increased proinflammatory conditions, other factors associated with metabolic dysregulation that occurs in maternal obesity—such as hyperglycemia, increased in insulin and leptin levels, mitochondrial dysfunction, and elevated oxidative stress—can create a complex and challenging environment for normal fetal development (Catalano et al. 2009; Costa et al. 2016). Mitochondrial dysfunction and inflammation are convergent intracellular changes, which can lead to cellular dysfunction and apoptosis (de Mello et al. 2018). Mitochondria function is highly responsive to inflammatory signals, and in turn, can release others mitochondrial components and trigger more inflammatory response and cell stress—creating a self‐reinforcing feedback loop (Zhang et al. 2010). Obesity itself is correlated to mitochondrial dysfunction in the central nervous system (to review check (de Mello et al. 2018; Schmitt et al. 2024; Schmitt and Gaspar 2023)). In MIA context, mice exposed to immune activation in pregnancy, the pups showed lower mitochondrial membrane potential, decreased ATP levels and increased release of free radicals in the brain, suggesting MIA can lead to mitochondrial dysfunction in offspring brain (Cieślik et al. 2023). Mice model of maternal exposure to HFD impaired mitochondrial dynamics on offspring brain, resulting by increased of mitofusin‐2 (Mfn2) protein content and decreased of dynamin‐related protein‐1 (DRP1) protein content, while mRNA expression levels of Mfn2 are reduced (Cardenas‐Perez et al. 2018). Furthermore, multi‐omics analyses revealed modified hippocampal transcriptomic profile, particularly in genes related with oxidative phosphorylation complex, in adults offspring from rodent mothers of HFD‐ induced obesity (Gauvrit et al. 2023). In the placentas from women with obesity, was observed a decrease in the activity of the antioxidant enzymes superoxide dismutase (SOD) and catalase, accompanied by increased levels in the nitrotyrosine residues, suggesting that the placenta antioxidant response of women with obesity is affected (Santos‐Rosendo et al. 2020).

In summary, all the inflammatory and metabolic changes that occurs in obesity during pregnancy can cross the blood placental barrier and influence the fetal brain inflammatory, mitochondrial function and metabolic profile, that can affect fetal and offspring brain development.

## 3 Neurodevelopmental Impact of Glucotoxicity and Lipotoxicity in Offspring of Obese Mothers

Obesity is associated with elevated circulating levels of glucose and free saturated fatty acids, and the excessive supply of these metabolites can lead to two detrimental conditions known as glucotoxicity and lipotoxicity (Poitout and Robertson 2002). Lipotoxicity refers to the detrimental effects of excess lipids, particularly saturated fatty acids, on cellular function and viability. In the brain, elevated levels of saturated fatty acids—commonly observed in the context of obesity and high‐fat diets—can disrupt neuronal homeostasis and contribute to neuroinflammation, mitochondrial dysfunction, oxidative stress, and endoplasmic reticulum (ER) stress (Cavaliere et al. 2019; Diaz et al. 2015; Gupta et al. 2012; Kwon et al. 2014; Schmitt et al. 2024; Yi et al. 2017). Additionally, saturated fatty acids may compromise the integrity of the BBB and interfere with insulin and leptin signaling, both of which are critical for central regulation of energy homeostasis and cognitive function (Benoit et al. 2011; Kanoski et al. 2010). Over time, these processes can result in behavioral and cognitive impairments, including increased anxiety, memory deficits, and susceptibility to others psychiatric disorders, as well as neurodegeneration.

Human placenta from mothers with obesity have higher clearance on free fatty acids (FFA) transport compared to lean mothers, suggesting the direct transport of FFA to the fetus is elevated in mothers with obesity (Hirschmugl et al. 2021). Also, human data of lipidomic analysis of the cord‐blood from mothers with obesity showed increased levels in saturated fatty acids (palmitate and stearate) compared to cord‐blood from lean mothers (Costa et al. 2016). High levels of triglycerides and cholesterol on mother's plasma is positively correlated with offspring plasma levels of triglycerides and cholesterol (Malti et al. 2014).

Animal studies, have demonstrated that maternal diet can influence the fatty acids composition of fetus brain lipids (Pavey and Widdowson 1980). In murine model, the lipidomic profile of offspring from free‐choice high fat‐high sugar diet fed mothers showed increased concentration of sphingolipids on frontal cortex and hippocampus, followed by enhanced gene expression levels of ceramide synthase 2 in the hippocampus (Santillán et al. 2025). Moreover, the sphingolipids enhanced concentration was also followed by cognitive impairment on pups from free‐choice high fat high sugar diet fed mothers (Santillán et al. 2025). Maternal HFD consumption is also linked with increase of lipid peroxidation on fetus brain, which was associated with synaptic impairment, in a mice model of obesity (Hatanaka et al. 2016; Tozuka et al. 2009). Thus, the increase of lipids on fetal brain can disrupt brain homeostasis.

Glucotoxicity is another harmful consequence that can arise in the context of obesity or diabetes, where abnormally high glucose levels may accumulate and exert toxic effects throughout the body. One of the most critical aspects of glucotoxicity involves the formation of advanced glycation end products (AGEs), which result from a hyperglycemic environment that induces irreversible, non‐enzymatic binding of glucose to molecules such as proteins. These AGEs are toxic, unstable, and highly reactive (Twarda‐clapa et al. 2022). Fluctuations in glucose levels—commonly observed in type 2 *diabetes mellitus*—can be change neuronal homeostasis and function, as demonstrated in in vitro studies using C6 astrocytes, as well as neuronal cell cultures (Gaspar et al. 2010, 2013; Hansen et al. 2012); as well as induced mitochondrial dysfunction, increased reactive oxygen species (ROS) and proinflammatory cytokine production, and impaired glutamate and glucose uptake in glial cells (Nokin et al. 2017; Peng et al. 2016; Quincozes‐Santos et al. 2017; Rivera‐Aponte et al. 2015).

Under glucotoxicity conditions there is an increase in methylglyoxal production, which is the main precursor of AGEs. Higher levels of methylglyoxal in maternal circulation can cross the placental barrier and lead to premature neurogenesis and decrease neural precursor cells, suggesting a neural impairment which can persist postnatally (Yang et al. 2016). As well, maternal high fat diet consumption can impair the glucose metabolism in fetus, particularly in the hypothalamus (Chen et al. 2014). The hypothalamic neurons from male pups from mothers fed HFD showed reduced response to hyperglycemia stimuli in vitro, even for the neuropeptide Y (NPY) neurons and for the pro‐opiomelanocortin (POMC) neurons, suggesting offspring hypothalamic glucose uptake was reduced by maternal HFD consumption, which can lead to hyperphagia and food imbalance (Chen et al. 2014). Additionally, insulin and leptin are known as the main anorexigenic signaling hormones and are essentials for glucose homeostasis (Varela and Horvath 2012). Embryos of HFD‐exposed mothers were hyperinsulinemic and hyperleptinemic but their intracellular signaling pathway was found to be disrupted in fetus's hypothalamus, with decreased expression of IRS‐2 and STAT‐3 (Gupta et al. 2009). This suggests that fetus from HFD mothers have resistance to insulin and leptin on hypothalamus and reflect in neuroendocrine alteration (Gupta et al. 2009). Furthermore, glucose transporters (GLUTs) are a family of transporter protein responsible for transport glucose to cell, which GLUT1 present in endothelial cells of the BBB, and GLUT3 present in the neurons. GLUT 4 is less abundant in brain, but is found in hippocampus, as well as in cerebellum, and is the insulin responsive glucose transporter. On maternal HFD model, the offspring on postnatal day (PD) 21 decreased mRNA expression of GLUT1 and GLUT4 on hippocampus (Abedi et al. 2024). At PD 180 (adult offspring), was observed decreased content of GLUT3 in the hippocampus of the offspring which continued eating HFD since birth (Abedi et al. 2024). Taken together, maternal HFD exposure can dysregulate brain glucose signaling, which can cause cerebral metabolic effects.

**FIGURE 1:**
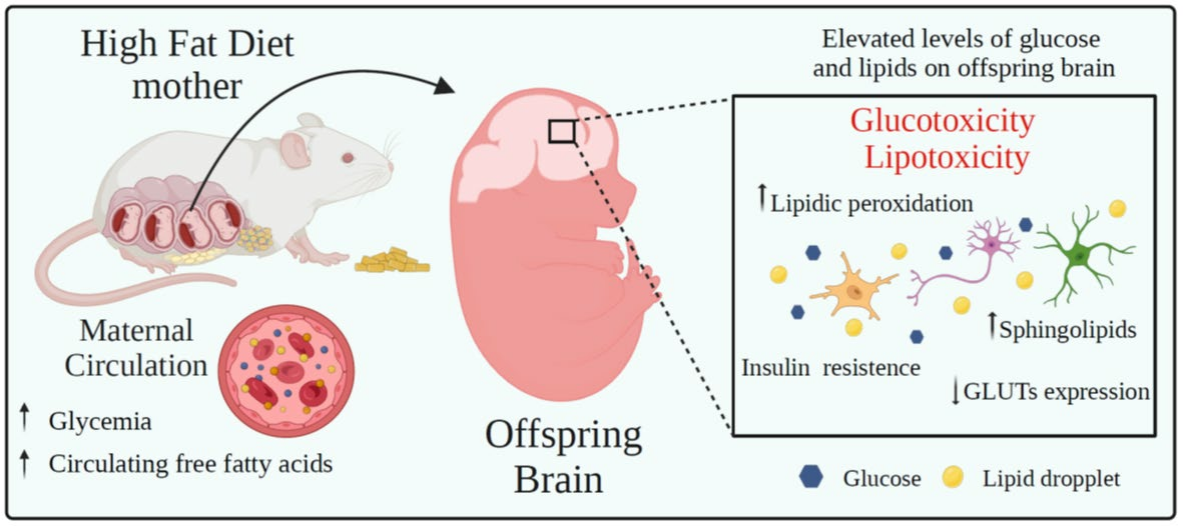
Maternal high‐ fat diet causes glucotoxicity and lipotoxicity in the offspring brain. Maternal obesity induced by high‐ fat diet elevates the circulating levels of glucose and free fatty acids. Increased levels of glucose and lipids can cross the placental barrier and reach the developing offspring brain, and trigger glucotoxicity and lipotoxicity conditions. These situations can disrupt brain cells homeostasis and impair neurodevelopmental processes. Figure created using BioRender.com.

In summary, maternal obesity, as well as the consumption of a high caloric diets (high fat or high sugar) may contribute to neurodevelopmental abnormalities in the fetal brain through both lipotoxic and glucotoxic mechanisms (Figure 1). These mechanisms involve a range of molecular and cellular pathways which can impact the offspring brain homeostasis and lead to functional impairment in brain activity.

## 4 Maternal Obesity and Its Influence on Offspring Neurodevelopment and Neurotransmission

Maternal obesity is increasingly recognized as a critical factor influencing fetal brain development, with long‐term consequences for offspring. In humans, newborns from mothers with obesity showed less cerebral white matter integrity (measured by fractional anisotropy), suggesting that maternal adiposity can have a negative impact on brain white matter development (Ou et al. 2015). Also, maternal high body‐mass index (BMI) is negativity correlated with cerebellum development at pregnancy (Koning et al. 2017). As well, maternal BMI is correlated with brain connectivity on neonatal thalamus, showing increased connectivity in left thalamus but decreased connectivity in frontothalamic region, suggesting maternal BMI is associated with brain circuit development, which can impact child cognitive, social, and behavioral capacities (Spann et al. 2020).

The hypothalamus is a brain region essential for regulating circadian rhythms, feeding, body temperature, whole‐body energy metabolism, and managing emotions. Hypothalamus has a regulatory function in controlling hunger by responding to peripheral anorexigenic signals (appetite reduction) and orexigenic signals (appetite stimulation), exerting potent effects on energy homeostasis (Varela and Horvath 2012). In the embryonic hypothalamus of Sprague–Dawley rats of HFD‐fed dams it was found an increase in the expression of NPY as well as in the number of orexigenic NPY expressing neurons (Poon et al. 2012). These orexigenic changes were also shown in another study with Sprague–Dawley embryos, that immunohistochemistry essay revealed increased immunoreactivity of AgRP/NPY neurons, while a decrease in immunoreactivity of α‐MSH in the cells, suggesting a suppression of anorexigenic signaling in the hypothalamus of fetuses from HFD fed mothers (Stachowiak et al. 2013). Changes in orexigenic and anorexigenic peptides, were maintained until 3 months old offspring of mothers fed with HFD (Ornellas et al. 2016). This increased NPY and decreased POMC was also followed by elevated SOCS3 and decreased JAK2/STAT3 phosphorylation, indicating central impairment of leptin signaling (Ornellas et al. 2016). In addition, rats from maternal overnutrition at first day of age decreased hypothalamic mTOR, pAMPK, and DNMT1 protein, which can impact neural progenitor cell proliferation and differentiation (Desai et al. 2016). At 6 months of age the offspring from maternal overnutrition increased AgRP/NPY and decreased POMC proteins in the arcuate nucleus of the hypothalamus, proposing that maternal overnutrition can modulate neuronal differentiation (Desai et al. 2016). The consumption of high fat high sucrose diet before pregnancy, induces ER stress in the hypothalamic arcuate nucleus POMC and AgRP neuronal population in the offspring at PD10 (Park et al. 2020). Also, maternal programming by HFD was capable of causing ER stress in the hypothalamus of adults offspring rats, which reflected in enhanced ER‐mitochondrial interaction and metabolic compromise in offspring (Cardenas‐Perez et al. 2018). In a murine model, offspring of females fed a HFD prior to gestation showed decreased expression of proliferative genes in the hypothalamus at embryonic day (ED) 13, specifically *Bub1b*, *Ki67*, and *Pcna* (Dearden et al. 2020). Hypothalamic transcriptome profile of adult offspring exposed to maternal obesity showed downregulated genes expression in pathways involved in oxidative phosphorylation, also indicating mitochondrial dysfunction on hypothalamus (Kulhanek et al. 2022). Taken together, maternal exposure to HFD in utero can disrupt hypothalamic cell homeostasis in the offspring and influence their energy metabolism in both the ihort and long‐term, primarily by affecting the neurons responsible for feeding behavior.

Hippocampus is the brain region fundamental for cognitive functions, such as memory, learning and spatial orientation. Moreover, it is one of the few regions in the adult brain capable to produce new neurons, as part of structural neuroplasticity (Leuner and Gould 2010). Hippocampus is densely rich in neurons that use glutamate as their primary neurotransmitter (McBain et al. 1999). MIA in animals models is also correlated with suppression of hippocampal postnatal neurogenesis, increased proinflammatory cytokines, reduced basal neurotransmission of dopamine and glutamate, and decreased levels of Gamma Aminobutyric acid (GABA) (Bilbo and Schwarz 2012; Meyer et al. 2006).

The consumption of high fat diet in a rodent model, before pregnancy increased the levels of extracellular glutamate in offspring hippocampus, accompanied by Vesicular glutamate transporter 1 (VGLUT1) up‐regulation, demonstrating that HFD can impact offspring glutamate homeostasis and synapses (Mizera et al. 2022). Maternal HFD consumption induces Glutamate Ionotropic Receptor AMPA Type Subunit 1 (GlutA1‐AMPA receptor subunit) hyperpalmitoylation (excessive chemical covalently attachment of palmitate to proteins) on offspring hippocampus, suggesting a posttranslational modifications on hippocampal glutamate receptors (Lin et al. 2021). Maternal overnutrition can also affect the expression of N‐methyl D‐aspartate receptor (NMDA) receptors on offspring hippocampus by enhancement of glutamate [NMDA] receptor subunit epsilon‐2 (GluN2B) protein levels in young adults rats (Mizera et al. 2022). The increase of GluN2B subunit on hippocampus seems to be negatively correlated to adult neurogenesis (Hu et al. 2008), suggesting it can be a possible mechanism of how maternal HFD can impact offspring neurogenesis in the offspring young rats (Mizera et al. 2022).

Brain‐derived neurotrophic factor (BDNF) is a protein essential for brain development, neuroplasticity and repair neurons. In the hippocampus, this protein plays a key role in learning, memory and mental health (Erickson et al. 2012). In female rats fed with saturated HFD, their adult offspring exhibited reduced levels of BDNF, nerve growth factor (NGF), and activity‐regulated cytoskeleton‐associated protein (Arc) both at mRNA and protein level in the hippocampus (Page et al. 2014). In 4‐week‐old mouse pups whose mothers consumed HFD, there was a reduction in hippocampal expression of *BDNF*, *cyclic AMP response element‐binding protein* (CREB), and *Grin2b*, alongside increased expression of DNA methyltransferase genes. These findings suggest that maternal exposure to a HFD may impair offspring neurodevelopment through gene hypermethylation (Yan et al. 2017). Additionally, adult offspring mice of HFD‐fed mothers showed decreased hippocampal BDNF protein levels, reduced 5‐HT_1A_ receptor protein expression, and elevated Mash1 protein levels, all indicative of disrupted hippocampal neurogenesis (Curi et al. 2021).

Epigenetics alter how gene are expressed without change the DNA code. In a Wistar rat model, it was reported an increased in hippocampal H4 histone acetylation at PD21 of offspring from HFD fed mothers, but decreased at PD50 (Gonçalves et al. 2017). Also it was observed that at ED17.5 male pups from high fat diet mothers enhanced binding of histone H3 lysine 9 acetylation at oxytocin receptor (OXTR) promoter compared to male embryos from control mothers (Glendining and Jasoni 2019). The females pups from HFD fed mothers had lower OXTR promoter histone H3 lysine trimethylation compared to female pups from control mothers (Glendining and Jasoni 2019). Those results show that maternal HFD consumption can induce epigenetic changes in hippocampal DNA histone binding in offspring differently, according to pup sex.

Dopamine is the neurotransmitter correlated to reward and motivation, being mainly produced by *substantia nigra* and ventral tegmental area (VTA). The dopamine neurons from VTA projects to hippocampus, nucleus accumbens (NAc), prefrontal cortex (PFC) and amygdala, being important to memory formation, emotional or motivational experiences and learning (Hou et al. 2024; Ikemoto 2007; Sayegh et al. 2024; Tsetsenis et al. 2023). In rodents, the offspring from mothers that consume HFD showed increased in the dopamine transporter (DAT) gene expression on VTA, NAc and PFC. The expression of the dopamine receptors D1, D2 and cAMP‐regulated phosphoprotein DARPP‐32 on NAc and PFC decreased in offspring from mothers that consume HFD, demonstrating maternal HFD consumption can alter dopamine circuits (Vucetic et al. 2010). Moreover, maternal HFD consumption during lactation changed the dopaminergic brain circuits by silencing dopaminergic midbrain neurons, attenuated synaptic connectivity with downstream projections sites and decreased dopamine release in striatum (Lippert et al. 2020). In the NAc, it was observed that male offspring from HFD fed mothers increased DAT and D2 receptor content, effect that was not observed in female offspring (Dias‐Rocha et al. 2023). In contrast, female offspring from HFD mothers show increased DARPP‐32 content in the NAc, whereas male offspring exhibit decreased levels (Dias‐Rocha et al. 2023). Taken together, these findings suggest that maternal HFD consumption can compromise the integrity of dopaminergic circuits in the offspring, with effects influenced by the sex of the pup, potentially leading to impaired reward processing and motivation.

GABA is the brain's primary inhibitory neurotransmitter and acts as regulator of neuronal excitability, being important for the balance between excitatory and inhibitory synapses. In mice, offspring from mothers fed a HFD presents an increase in the anxious‐like behavior and has an increased expression of GABA_A_ alpha2 receptor on ventral hippocampus compared to offspring from lean mothers (Peleg‐Raibstein et al. 2012). Western diet consumption by mothers induced in the hypothalamus of the offspring after weaning a decreased in GABA_A_ alpha5 subunit and an increased in GABA_A_ alpha1 in the NAc and VTA (Paradis et al. 2017), suggesting a remodeling of GABA neurotransmission.

Serotonin (5‐hydroxytryptinamine, 5‐HT) is the neurotransmitter that plays a key role in regulating mood, emotion, sleep, appetite, digestion and even memory. Serotonin is 90% synthesized on gut by enterochromaffin cells and the remaining pool is produced by the brain specially on raphe nuclei, where the amino acid tryptophan receives a hydroxyl group and forms the intermediate 5‐hydroxytryptophan, reaction that is catalyzed by tryptophan hydroxylase 2 enzyme (TPH2) (Watts et al. 2012). HFD consumption disrupt serotoninergic system by altering serotonin synthesis, transport, receptor signaling and gut‐brain interactions (Chakraborti et al. 2021; Hoch et al. 2023; Huang et al. 2004; Watanabe et al. 2016). In maternal HFD model of nonhuman primate, was observed on offspring the increased expression of TPH2 on rostral raphe, followed by increased expression of 5‐HT_1A_ autoreceptor (Sullivan et al. 2010). Murine models of HFD also showed impairment on serotoninergic circuit in brain offspring. In the offspring of mice fed a HFD during pregnancy and lactation occurs an increased protein levels of TPH2 on hippocampus (Dias et al. 2020). In the PFC occurs an increased levels of 5‐HIIA (serotonin metabolite), which reflected increased 5‐HIIA/5‐HT ratio, indicating an increased serotonin metabolism (Moreton et al. 2019). In male offspring mice born from mothers that fed a HFD, present a decreased expression of 5‐HT_2C_ receptor in PCF, NAc and striatum at PD28 but at PD63 occurred increased in the 5‐HT_2C_ receptor levels in NAc and striatum (Gawlińska et al. 2021). All together these studies point the evidence that the consumption of HFD during gestation and lactation disturb the GABAergic serotoninergic systems in brain offspring.

In summary, maternal exposure to HFD has been shown to adversely affect neurodevelopment and the organization of neurotransmitters circuits in the offspring. This dysregulation involves multiple neurotransmitters systems, spans various brain regions, and is associated with alteration on key molecular pathways, including those regulating neurotransmitter synthesis, receptors expression and signaling efficiency (Table 3). Collectively, these neurobiological disrupts may impair fetal neurodevelopment disorders and neuropsychiatric conditions associated with maternal obesity.

## 5 Maternal High‐Fat Diet and Its Impact on Offspring Neuroglial Cells

The consumption of HFD by the mother during pregnancy can significantly influence the development and function of glial cells in the offspring's brain. Glial cells are the non‐neuronal cells of nervous system, including astrocytes, microglia, and oligodendrocytes. Glial cells play crucial roles in maintaining neural homeostasis, supporting neuronal function, and modulating inflammatory responses. In different rodent models, exposure to excessive dietary fats in utero has been shown to alter glial cell activation and morphology, potentially leading to neuroinflammation and impaired neural connectivity (Davis and Mire 2021; Hatanaka et al. 2016; Maldonado‐Ruiz et al. 2019). These changes may contribute to long‐term cognitive and behavioral deficits in the offspring, highlighting the importance of maternal nutrition for healthy brain development (Niculescu and Lupu 2009).

Microglia are the immune cells from the brain which function as sensors for environmental changes. However, immature microglia activation at early life can lead to persistent changes in microglia function, resulting in long‐term neural and cognitive dysfunction (Davis and Mire 2021). Upon exposure to a wide range of stimuli, microglia become rapidly activated, undergoing to morphological transformation, from ramified into an ameboid phenotype, followed by upregulation of various surface molecules associated with immune activation (Colonna and Butovsky 2017). In this process, microglia can assume an inflammatory response of M1 or M2 type, but the overactivation to M1 (microgliosis) can be proinflammatory and neurotoxic (David and Kroner 2011). Maternal immune activation disrupts epigenetic regulation in offspring microglia (Mattei et al. 2017). In nonhuman primates, female macaques consuming a Western‐style diet (WSD) high in saturated fats and sugars produced offspring that, at 13 months old (equivalent to 3–4 human years), showed decreased microglial cell counts in the amygdala (Dunn et al. 2022). Additionally, the number of microglia was correlated with both maternal WSD consumption and maternal adiposity (Dunn et al. 2022). A rodent model of HFD induced obesity throughout gestation and lactation showed increased density of microglia in offspring hippocampus (Ojeda et al. 2023), as well as changed microglia morphology even in males and females offspring at PD30, characterized by increased solidity and having shorter branch length (Bordeleau et al. 2020). At 6 weeks old, mice from HFD mother showed increased microglia density and microglia soma in hippocampus, altering the morphology and the number of microglia (Shiadeh et al. 2024). In rodents model of female Wistar rats which received cafeteria diet with 49% of fat before get pregnant, the pups at 8 weeks of age had an increased hypothalamic microglial activation (Maldonado‐Ruiz et al. 2019). Thus, maternal consumption of hypercaloric diets (cafeteria and HFD) can induce alterations on microglia morphology and increases the number of activated microglia (Table 3).

Microglia cells also are essentials for synaptic regulation, actively participating in brain plasticity and the process of synaptic pruning. The offspring of mothers that consume HFD before mating, exhibited a decrease number of mature lysosomes on microglia cells, increased microglia interaction on synaptic contact, decreased insulin like growth factor 1 (*Igf1*) gene expression, which is a growth factor secreted by microglia, and changed myelin organization, suggesting maternal HFD can modified myelination and impact microglia function, through microglia activation (Bordeleau et al. 2021). Moreover, microglia seem to interact directly to AgRP neurons and permanently alter the innervation on paraventricular hypothalamic nucleus after maternal HFD exposure (Mendoza‐Romero et al. 2025). Also, offspring from HFD mother at PD16 showed reactive microglia with enhanced hypothalamic phagocytic activity and increased internalized PSD‐95 protein, suggesting that microglia from HFD mothers offspring increased synaptic engulfment (Valdearcos et al. 2025). In addition, maternal high fat diet exposure also seems to modify offspring cerebrovascular system by increasing the microglia‐blood vessel proximity and enhanced microglial‐synapses interaction, which can be a consequence of exacerbated inflammation on offspring's brain (Bordeleau et al. 2022).

Astrocytes are the most abundant glial cells in the CNS and are responsible for neuronal support and maintenance, regulate neurotransmission, modulate synapse and participate in immune response. Also, their localization on BBB make those cells sensitive to metabolites and inflammation biomolecules (Abbott et al. 2006). Obesity induced by the consumption of HFD during pregnancy leads to increased proliferation and number of astrocytes on offspring hypothalamus (Kim et al. 2016). Adult offspring of mice whose mothers received a supplement of high‐sucrose soft drink and chocolate did not show changes in Glial Fibrillary Acidic Protein (GFAP)‐positive cells (a marker of astrogliosis) in the hypothalamus. However, when the offspring were exposed to the same diet, there was an increase in GFAP‐positive cells (Kjaergaard et al. 2017). The hypothalamic transcriptome of mice pups at PD15 (lactation peak) from HFD mothers did not change the astrocyte transcriptome profile compared to controls (Huang et al. 2024). But the cell‐to‐cell prediction interaction based on transcriptome showed increased interaction between hypothalamic AgRp/NPY neurons (orexigenic neurons) and astrocytes in then offspring from HFD mothers, indicating it can be an important interaction to obesity and metabolic disorders development (Huang et al. 2024).

**FIGURE 2:**
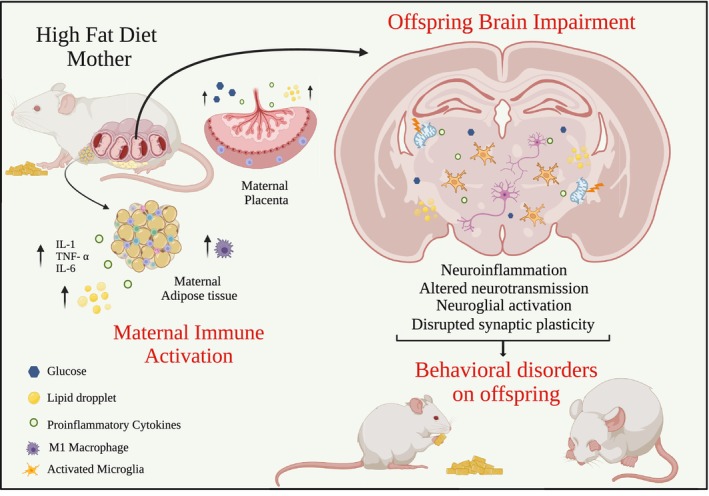
Maternal high fat diet consumption and impact in the offspring brain. Maternal high fat diets consumption increases the circulating levels of maternal proinflammatory cytokines, lipids and glucose which can traverse the placental barrier and directly affect the fetal development. The elevated proinflammatory cytokines, lipids droplets and higher glucose concentrations arrive to offspring brain and compromise cerebral homeostasis. This impairment on offspring brain results in neuroinflammation, altered neurotransmission, neuroglial activation and disrupted synaptic plasticity. Collectively, all these pathophysiological changes can contribute to the development of behavior disorders on offspring, which can manifest in short‐term and long‐term. Figure created using BioRender.com.

Thus, maternal HFD consumption can affect the neuroglial cells and influence the development and the function of offspring brain, which might lead to the development of obesity, metabolic syndrome, and also neurodevelopment disorders (Figure 2).

## 6 Impact of Maternal Obesity on Offspring Brain Disorders

Perinatal exposure to a maternal HFD and maternal obesity has been linked to a range of neurobiological alterations and subsequent behavioral changes in offspring, observed across both childhood and adulthood (Edlow 2017). Studies using humans (Table 1) and animal models (Tables 2 and 3), including rodents and nonhuman primates, have been crucial for elucidating the mechanisms by which maternal obesity and a HFD consumption affect offspring development, and consequently childhood obesity, metabolic disorders, and psychiatric disorders (Gao et al. 2013; Hochner et al. 2012; Nivins et al. 2024; Whitaker 2004). Research findings indicate that exposure to these conditions during gestation can induce significant alterations in brain development, disrupting key neurotransmitter systems such as the serotonergic and dopaminergic pathways, as well as promoting neuroinflammation and affecting processes like myelination (Bordeleau et al. 2021; Frankowska et al. 2023; Graf et al. 2016; Sullivan et al. 2010; Thompson et al. 2018). These neurobiological changes result in marked behavioral modifications in the offspring, including increased anxiety, cognitive deficits, alterations in social behavior, and heightened sensitivity to rewards such as palatable foods and drugs of abuse, and, in some instances, behaviors that parallel those associated with neurodevelopmental disorders (Bordeleau et al. 2021; Frankowska et al. 2023; Peleg‐Raibstein et al. 2016; Sasaki et al. 2014; Wu et al. 2013).

Increased anxiety‐like behavior is one of the most frequently reported behavioral outcomes. It was observed that maternal exposure to a HFD led to elevated anxiety‐like behavior in nonhuman primate offspring during infancy and juvenile stages, respectively, maybe due to altered expression of tryptophan hydroxylase 2, the serotonin transporter (SERT), and the 5‐HT_1A_ receptor (Sullivan et al. 2010; Thompson et al. 2018). Similarly, it was reported that adult rat offspring of HFD‐fed dams exhibited more anxiogenic behavior in the open field test and the elevated plus maze. An additional noteworthy finding comes from a subsequent study by the same group which showed that adolescent offspring exposed to maternal HFD during lactation displayed decreased anxiety‐like behavior, suggesting that the outcomes of early‐life programming may vary depending on the developmental window targeted (Sasaki et al. 2014). Sivanathan and colleagues further found that in adult rats with chronic HFD consumption exhibited increased anxiety‐like behavior, indicating that a direct effect of HFD treatment, beyond perinatal programming, may also be involved (Sivanathan et al. 2015). In nonhuman primates early nutritional intervention (switching to a control diet at weaning) was not sufficient to fully reverse the maternal HFD‐induced increase in anxiety, emphasizing the difficulty of mitigating the effects of adverse programming once established (Thompson et al. 2018).

Cognitive difficulties are also a common outcome of gestational obesity, and the dopaminergic system—which plays a critical role in regulating motivated behaviors, reward processing, and cognition—is likewise affected by maternal high fat diet programming. Maternal obesity in rats, induced by either a high fat or a high‐reward/high‐fat diet, led to deficits in reversal learning in adult offspring, along with notable disruptions in striatal dopamine regulation (including alterations in dopamine levels, dopamine metabolites, D2 receptor expression, and dopamine transporter activity) (Wu et al. 2013). Offspring of minipigs born to mothers fed a Western diet scored higher than those of control mothers in tests of working and reference memory, that might reflect enhanced cognitive abilities within the context of the task or greater food‐related motivation, despite observed detrimental effects on the hippocampus (Val‐Laillet et al. 2017). On male offspring mice born to HFD‐fed mothers during pregnancy has altered novel object recognition behavior, suggesting impaired memory function, that could be linked to reduced myelination in the medial cortex (Graf et al. 2016).

Changes in social behavior has also been reported in the offspring of mothers fed with a HDF prior and during gestation. Female mouse pups exposed to a maternal HFD, has social withdrawal which could be reversed through nutritional intervention during lactation (Kang et al. 2014). Meanwhile, maternal HFD in mice led to social impairments in offspring through alterations in the gut microbiome (Buffington et al. 2016). Notably, co‐housing with pups from mothers fed a standard diet or treatment with the bacterial strain *Lactobacillus reuteri* —which was reduced in HFD‐exposed offspring—was sufficient to reverse the social impairments (Buffington et al. 2016). The gut microbiome has emerged as an important mediator of the effects of maternal diet on offspring. Maternal HFD induced gut dysbiosis in mouse offspring, which was causally linked to the observed social behavior deficits in offspring (Buffington et al. 2016). It is important to note the interactions and existence of sex‐specific and developmentally timed effects. Dietary intervention during lactation was more effective at reversing social deficits and neuroinflammation in females than hyperactivity in males (Kang et al. 2014). Meanwhile, it was also reported reduced anxiety during adolescence in HFD‐exposed mice, in contrast to the increased anxiety observed in adulthood (Krishna et al. 2016).

Reward sensitivity, including the drive to obtain palatable foods and drugs of abuse, is also influenced by perinatal exposure to an HFD. It was demonstrated that overfed mouse offspring consumed more alcohol, showed increased sensitivity to amphetamines, an enhanced conditioned preference for cocaine, and a preference for sucrose and HFD (Peleg‐Raibstein et al. 2016). These findings indicates that obesity or HFD consumption during gestation can reprogram the fetal reward system that may increase vulnerability to addictive behaviors and obesity itself later in life.

Other studies have identified behaviors that mimic those observed in neurodevelopmental disorders, such as autism spectrum disorder (ASD) and hyperactivity related to Attention‐Deficit/Hyperactivity Disorder (ADHD). It was reported sociability deficits in offspring of HFD‐fed females and hyperactivity in male offspring exposed to maternal HFD (Kang et al. 2014). Post‐weaning HFD in primates led to an increase in stereotypic behavior (similar to ASD) (Thompson et al. 2018). In humans, a meta‐analysis showed that maternal pre‐pregnancy overweight and obesity (estimated by BMI) is related with an elevated risk of ADHD on offspring (Li et al. 2021).

Taken together, maternal obesity or exposure to a HFD during development has a significant impact on offspring brain functions, increasing their susceptibility to anxiety, cognitive impairment, and altered social behaviors, with potential implications for brain health and lifelong predisposition to obesity. The persistence of these effects throughout the offspring's lifespan, even when they are switched to a healthy diet after weaning—as observed in several (Kang et al. 2014; Krishna et al. 2016; Peleg‐Raibstein et al. 2016)—highlights the critical importance of the perinatal developmental window.

## 7 Conclusion

In summary, the studies described in this manuscript provide robust evidence that maternal obesity, and the consumption of HFD and/or high sucrose diets during critical periods of development, such as pregnancy and lactation, have profound and lasting detrimental effects on the brain, behavior, and metabolism of offspring. Neurobiological changes, including dysfunction in the neurotransmission systems, neuroinflammation, and impaired myelination and plasticity, with adverse behavioral phenotypes such as anxiety, cognitive deficits, social problems, and increased vulnerability to addiction. Metabolic programming also predisposes offspring to obesity and related metabolic disorders.

This has public health implication, since the high prevalence of obesity and the widespread consumption of high fat diets in modern societies, there is growing concern about their impact on the mental and metabolic health of future generations. Strategies aimed at improving maternal nutrition before and during pregnancy and lactation, as well as early‐life interventions in offspring, may be crucial in breaking the intergenerational cycle of obesity and related disorders. Understanding these mechanisms and identifying mediating factors open pathways for the development of new preventive and therapeutic strategies. Maternal nutritional health is, therefore, a fundamental pillar for the health and well‐being of future generations.

In addition, despite the growing number of studies on maternal obesity and its impact on offspring, there are still noticeable gaps in the literature, particularly regarding differences in neurodevelopment and behavior according to offspring sex from mothers with obesity or fed a high caloric diet during pregnancy. Further research is needed to achieve a deeper understanding of behavioral and brain development variations related to offspring sex.

## Author Contributions

**Luisa O. Schmitt:** conceptualization, writing – original draft, methodology, writing – review and editing, investigation. **Giuseppe Faraco:** writing – original draft, writing – review and editing. **Tamires S. Stivanin:** writing – original draft, writing – review and editing. **Joana M. Gaspar:** conceptualization, investigation, funding acquisition, writing – original draft, writing – review and editing, supervision.

## Funding

Tamires S. Stivanin was financed by the Fundação de Amparo à Pesquisa e Inovação do Estado de Santa Catarina (FAPESC). Giuseppe Faraco was financed by the Fundação de Amparo à Pesquisa e Inovação do Estado de Santa Catarina (FAPESC), project TO number: 2024TR001581 (EDITAL DE CHAMADA PÚBLICA FAPESC N°09/2024 − Mulheres + Pesquisa 1°edição). Joana M. Gaspar was financed by Fundação de Amparo à Pesquisa e Inovação do Estado de Santa Catarina (FAPESC), project TO number: 2024TR001581 (EDITAL DE CHAMADA PÚBLICA FAPESC N°09/2024 − Mulheres + Pesquisa 1° edição), and project TO number: 2024TR002262 (EDITAL DE CHAMADA PÚBLICA FAPESC N°21/2024 − Programa de Pesquisa Universal); and Brazilian Federal Agency—Fundação Coordenação de Aperfeiçoamento de Pessoal de Nível Superior (CAPES).

## Conflicts of Interest

The authors declare no conflicts of interest.

## Data Availability

The authors have nothing to report.
